# Cystitis glandularis with concomitant Crohn’s disease leading to a paroxysm of Crohn’s disease with ulcerated external iliac vessels

**DOI:** 10.1186/s12894-024-01470-3

**Published:** 2024-04-17

**Authors:** Wu Ronghua, Zheng Ji, Liu Gang, Zhang Yun, Nie Xubiao

**Affiliations:** 1 Department of Urology, Second Affiliated Hospital of Army Medical University, Chongqing, 400037 China; 2 Department of Intensive Care Unit, Second Affiliated Hospital of Army Medical University, Chongqing, 400037 China; 3 Department of Cardiovascular, Second Affiliated Hospital of Army Medical University, Chongqing, 400037 China; 4 Department of Gastroenterology, Second Affiliated Hospital of Army Medical University, Chongqing, 400037 China

**Keywords:** Cystitis glandularis, Crohn’s disease, Vesicovaginal fistula repair, Ulcerated external iliac vessels, Serious complication

## Abstract

•we report the case of a 36-year-old female patient who presented to our hospital with a diagnosis of cystitis glandularis manifesting as a vesicovaginal fistula. She underwent cystoscopic biopsy at a local hospital, but anti-inflammatory treatment was ineffective, and the patient was experiencing low urination frequency and urgency, as well as pain. The patient underwent laparoscopic repair of a cystoscopy-confirmed vesicovaginal fistula. After surgery, the patient experienced a paroxysm of Crohn’s disease with multiple small bowel fistulas and erosion of the external iliac vessels that ruptured to form an external iliac vessel small bowel fistula. The fistula was confirmed by surgical exploration, and the patient eventually died.

## Introduction

Crohn’s disease is a major inflammatory bowel disease. This chronic condition lacks a medical cure and commonly requires a lifetime of care and medication [1]. Crohn’s disease is characterized by an unknown pathogenesis, lack of a diagnostic gold standard, unsatisfactory treatment, and poor prognosis [2] and is easily missed by nongastroenterologists in patients with atypical clinical symptoms [3]. Clinical complications such as intestinal fistulas and intestinal obstructions are common [4], and when chronic intestinal inflammation is inadequately controlled, progressive and irreversible bowel damage occurs. The potential complications of this progressive damage, such as bowel perforation, are serious [5]. Cystitis glandularis is a more common inflammatory disease in urology [6] and most often requires cystoscopy and pathological examination for diagnosis [7]. The co-occurrence of these two diseases has not been reported previously. The case reported here is a very rare case of cystitis glandularis combined with Crohn’s disease, which eroded the external iliac vessels and eventually led to the patient’s death.

## Case report

**Fig. 1:**
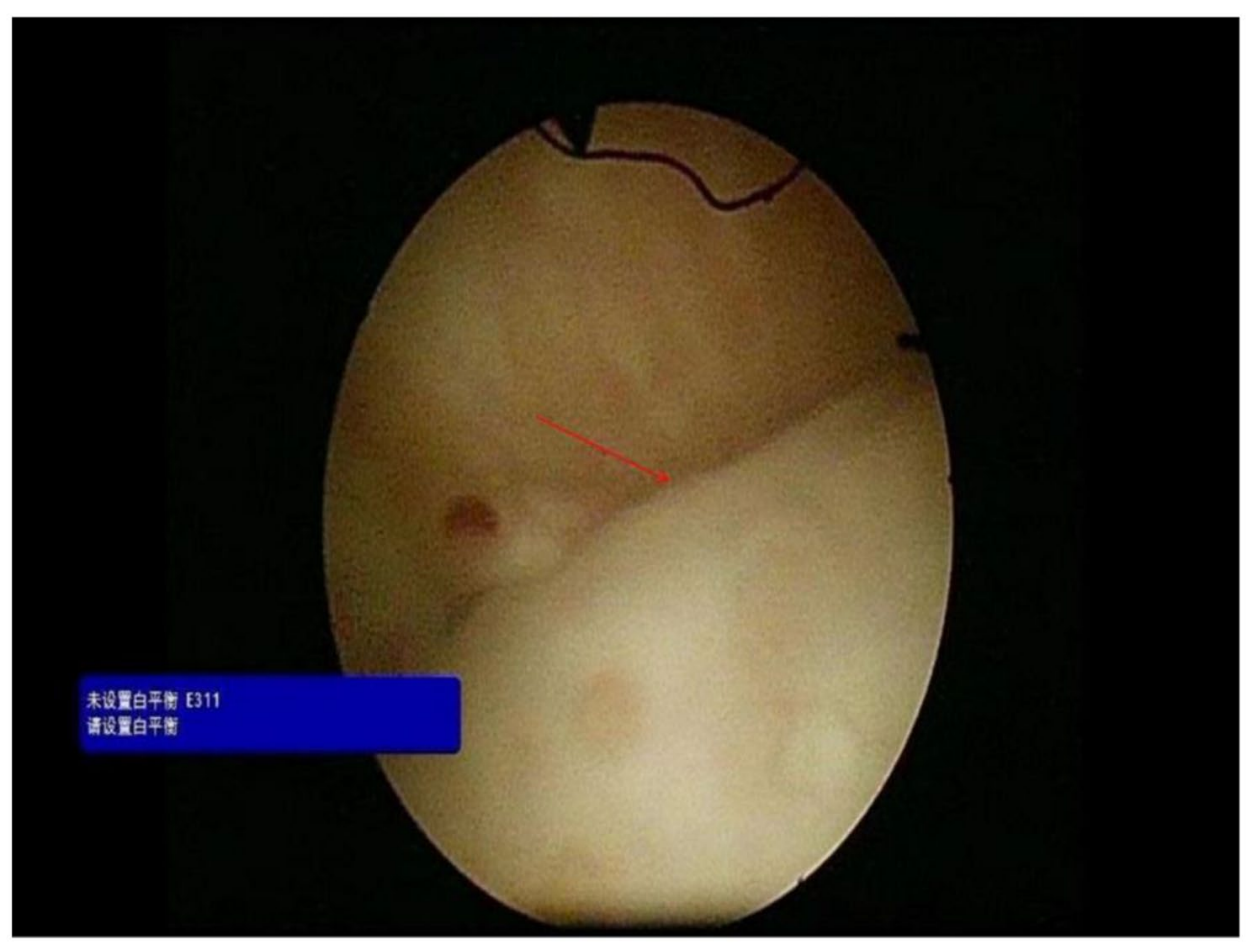
Cystoscopic visualization of a vesicovaginal fistula (red arrow)

**Fig. 2:**
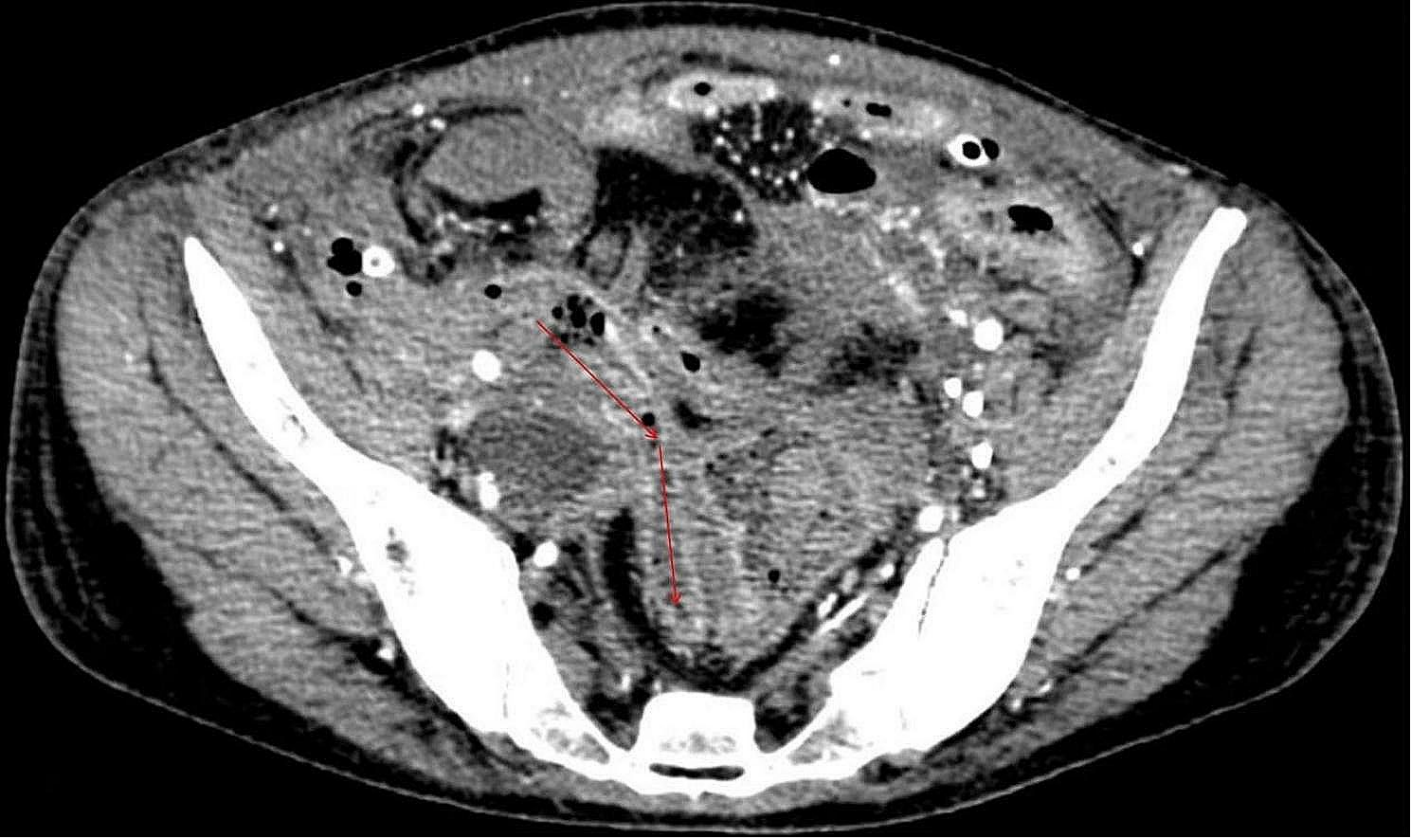
Computed tomography image showing an intestinal fistula (red arrow)

**Fig. 3:**
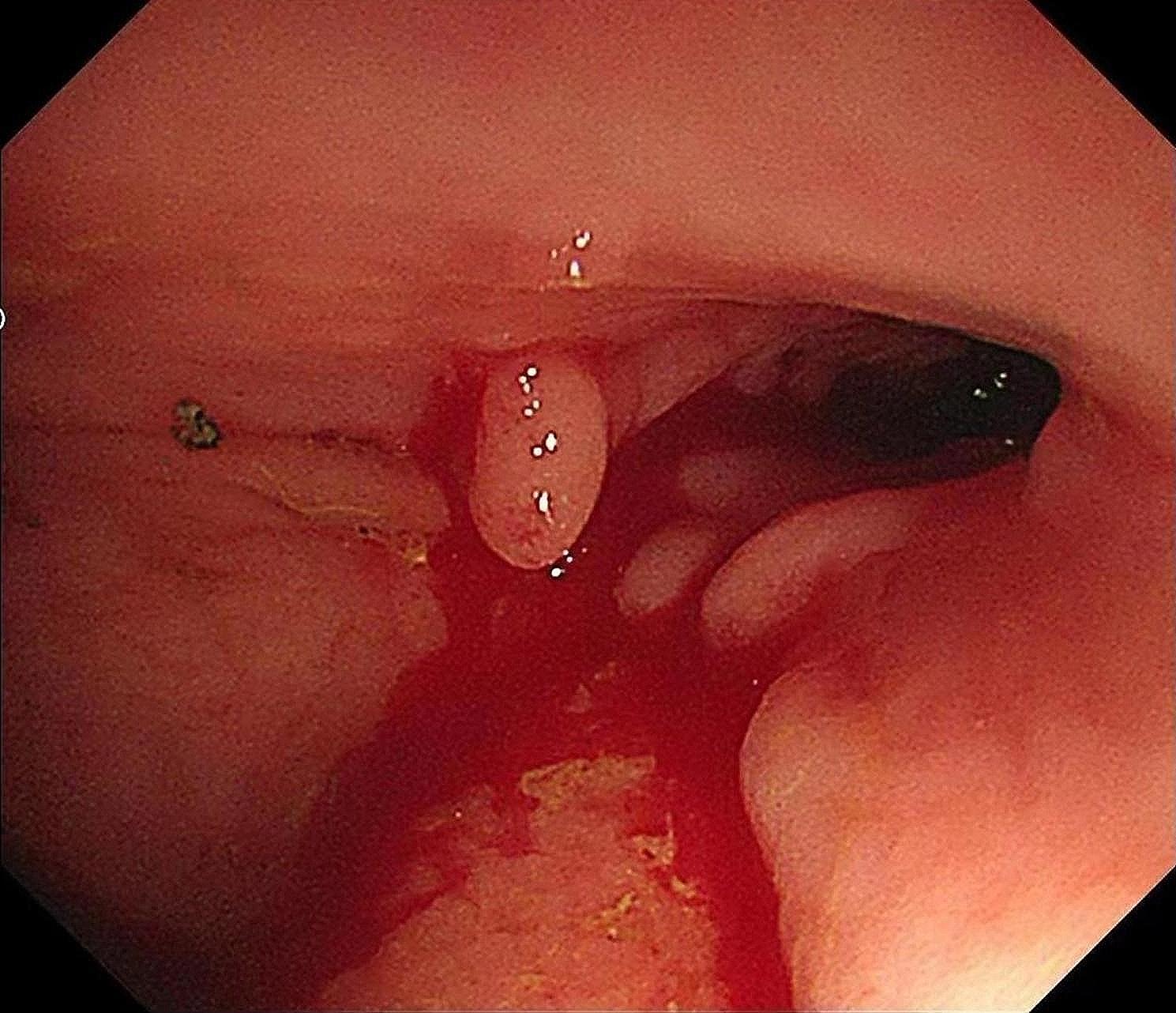
Small bowel fistula visible on colonoscopy

**Fig. 4:**
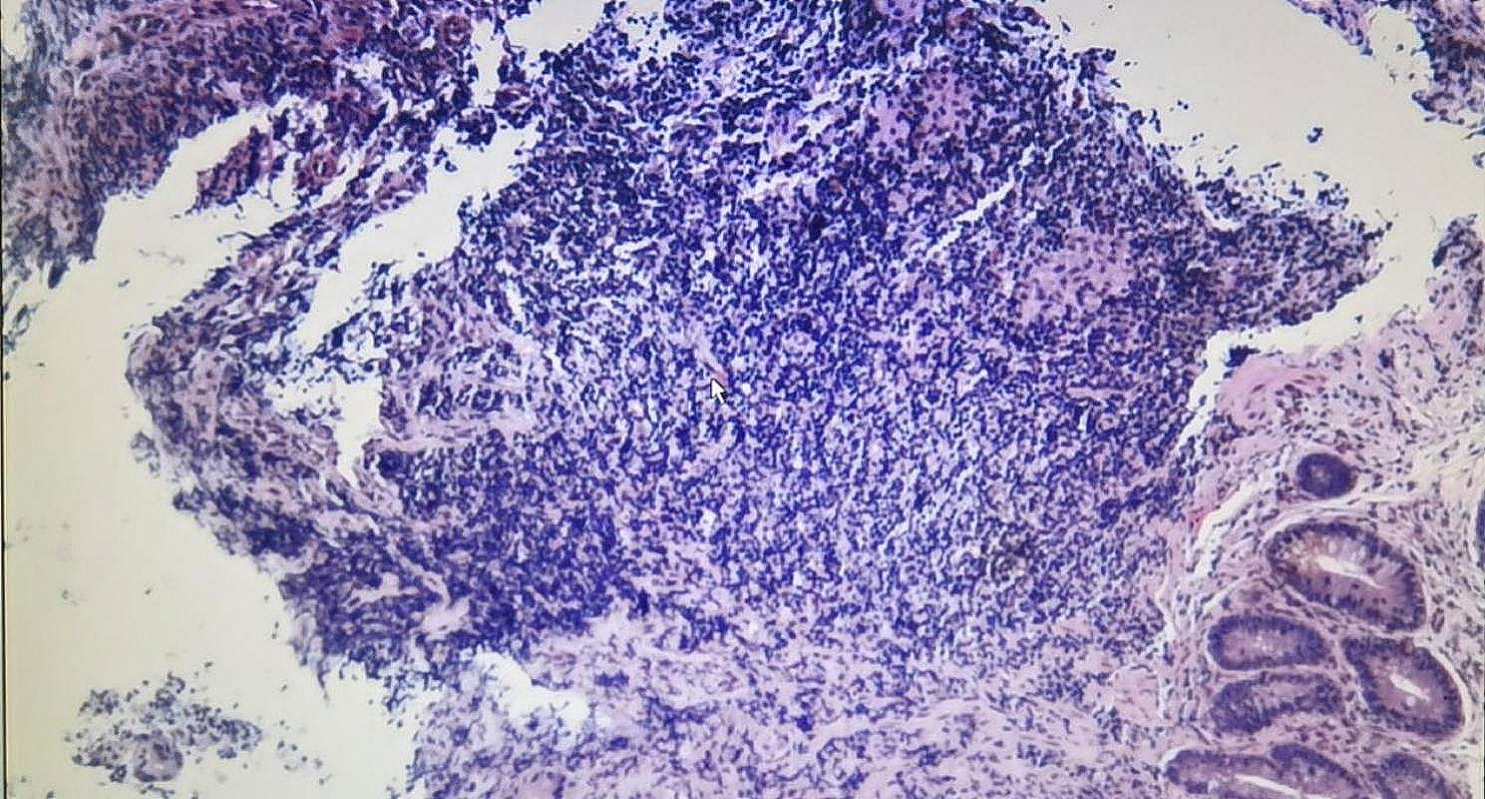
Multiple ulcers of the intestinal mucosa with lymphocytic, histiocytic and plasma cell infiltrates

The patient was a 36-year-old female with normal growth, a relatively lean body mass (BMI, 19.5), and a normal appetite. She was married, did not have children and had no history of conception. Her parents and younger brother were healthy. She had a history of chronic abdominal pain for more than 10 years and presented with intermittent episodes. She was not formally treated but was diagnosed with gastritis by her family doctor, and her symptoms were relieved with oral acid suppressants. She had had irregular stools for more than 10 years, had no bloody stools, had lost approximately 3 kg in the last 10 years, and had a history of laparoscopic appendectomy 10 years prior and perianal abscess incision 8 years prior. She was diagnosed with adenoid cystitis (self-reported, with no specific pathology report available) after a cystoscopic biopsy at a local hospital. Urine discharge from the vagina occurs approximately 1 week after cystoscopy, regardless of position, and patients come to our hospital for this reason. Cystoscopy was performed in our department to confirm a vesicovaginal fistula, and a site of leakage in the posterior wall of the bladder, approximately 1 cm in size, was observed (Fig. 1). During cystoscopy, irrigating fluid was observed flowing out of the vagina. Methylene blue was observed to flow out of the vagina after injection, and some follicle formation was observed in the bladder triangle, as well as a degree I prolapse in the anterior wall of the vagina. Follicle biopsy confirmed the diagnostic histopathological features of inflammatory cell infiltration with interstitial oedema. Routine urine test results were as follows: urine leukocytes, 2+; and erythrocytes, 3+. Other test results were normal. Laparoscopic vesicovaginal fistula repair was performed. Severe intra-abdominal bowel adhesions were observed intraoperatively and were most obvious in the pelvis. The bladder leak was completely resolved during the operation. After the operation, the bladder was kept at low pressure and rehydrated; the patient received a small amount of water 6 h after the operation and gradually resumed her diet. Two stools of normal colour and nature were passed after the operation, and on the 5th day after the operation, there was a sudden onset of bloody stool. The volume was approximately 500 ml, and the blood was fresh. Blood transfusions and other treatments were administered. Computed tomography suggested a possible intestinal fistula (Fig. 2) and a suspected breach of the external iliac vessel. A rupture was noted in the descending colon with direct access to the small intestine, and a small colonic fistula was considered (Fig. 3). Immediate emergency dissection revealed multiple fistulas in the sigmoid colon, descending colon, and small intestine and a ruptured sinus tract between the ascending colon and the right external iliac vessel, approximately 0.5 cm in diameter, through which blood entered the ascending colon, with soft tissue and vessel walls surrounding the right external iliac vessel; the fistulae could not be closed using conventional sutures. The vascular surgeon repaired the external iliac vessels using vascular spacers, and the general surgeon performed intestinal adhesion release, small bowel single-lumen fistula repair, intestinal sinus tract repair, and bowel resection. The entire exploratory surgery resulted in approximately 3000 ml of blood loss, and the patient was admitted to the intensive care unit. Postoperative pathology revealed acute and chronic inflammation invading the entire intestinal wall, with multiple ulcers in the intestinal mucosa with lymphocytic, histiocytic and plasma cell infiltration, consistent with Crohn’s disease (Fig. 4). The patient was given a blood transfusion, fasted, and received electrolyte stabilization, nutritional support, hormone supplementation, anti-inflammatory drugs, and infliximab (a human-mouse chimeric monoclonal antibody that binds to both soluble and transmembrane forms of TNF-α with high affinity), which inhibits the binding of TNF-α to its receptor and renders it inactive. The biological activities of TNF-α include the induction of proinflammatory cytokines such as IL-1 and IL-6 and increased endothelial permeability and expression of adhesion molecules by endothelial cells and leukocytes to enhance leukocyte migration. An initial dose of 5 mg/kg was given, followed by the same dose at weeks 2 and 6 after the first dose and every 8 weeks thereafter; for patients who exhibit a poor response, dose adjustment up to 10 mg/kg may be considered. The patient underwent additional treatments in the intensive care unit, but she experienced late septic shock and multiorgan failure. Ten days after the operation, another episode of bloody stool of approximately 1000 ml occurred, and approximately 2000 ml of fresh blood was drained from the abdominal drainage tube. The family declined further treatment; the patient was discharged, and she died 1 day later at home.

## Discussion

Crohn’s disease is a very common inflammatory disease, and as the disease progresses, it can easily endanger the patient’s life. Its pathogenesis and mechanisms are still unclear [8]. The common suspected causative factors and pathogenesis currently include geographical and food factors, intestinal cell injury, intestinal flora dysbiosis, pathophysiological changes in the intestinal mucosa or related tissues, and immune dysfunction [9]. A gold standard method for diagnosis is lacking, and diagnosis currently requires comprehensive and integrated analysis of symptoms in combination with the findings of relevant examinations, mainly colon features on endoscopy and histopathological characteristics. Crohn’s disease is characterized by transmural inflammation, and intestinal ulcers penetrating the serosal layer can form intestinal fistulas, which can be categorized as extraintestinal and intraintestinal fistulas according to their location; intraintestinal fistulas include enteroenteric fistulas and intestinal vesicovaginal fistulas [10].

Cystitis glandularis is a chronic inflammatory disease of the bladder caused by epithelial metaplasia [11]. There are three theories regarding the pathogenesis of cystitis glandularis, namely, the embryonic origin theory, the Pund degeneration theory and the epithelial metaplasia theory [12]. Epithelial metaplasia is widely accepted as a defence mechanism in which migratory metaplastic epithelium becomes glandular epithelium under the action of chronic irritants and achieves self-protection by secreting mucus [13]. Chronic irritants include but are not limited to infections, obstructions (female urethral syndrome, prostatic hyperplasia, urethral strictures, etc.), physical irritants (stones, foreign bodies), and chemical carcinogens. The clinical manifestations are mostly nonspecific, such as varying degrees of discomfort, urinary frequency, difficulty in holding urine, and lower abdominal distension. In severe cases, acute urinary retention, bladder contracture and upper urinary tract fluid accumulation may occur [14]. The confirmation of the diagnosis is mainly based on cystoscopy and pathological examination, and biopsy results are the gold standard for the diagnosis of cystitis glandularis [15]. There have been no reports of a vesicovaginal fistula after a cystoscopic biopsy in the literature on cystitis glandularis, which does not affect the bladder muscle. Vesicovaginal fistulas occur mainly after hysterectomy, caesarean section, radiotherapy and pelvic surgery [16] and are mainly caused by local ischemia and fibrosis. The cause of the vesicovaginal fistula in this patient is unclear; cystitis glandularis mainly occurs in the mucosal layer of the bladder, and further research is needed to determine the mechanism by which the tissue between the muscle layer of the bladder and the vagina is destroyed.

Crohn’s disease and cystitis glandularis in this patient were confirmed by pathology, and our searches of the PubMed® and Sci-Hub® databases using the two keywords “cystitis glandularis” and “Crohn’s disease” failed to retrieve any relevant studies. To identify common causative genes or signalling pathways for both diseases, considering that both diseases are correlated with tumorigenesis, we conducted bioinformatics-related searches through six databases, namely, TCGA, GEO, ICGC, HPA, CGGA, and DisGeNET [17], and failed to find common causative genes or signalling pathways. Considering that both cystitis glandularis and Crohn’s disease are also inflammatory diseases [18, 19], we searched the NCRI database with the keywords “Crohn’s disease” and “cystitis glandularis” [20], and no common inflammatory causative factors were found. In terms of potentially relevant biomarkers, we searched the SC2disease database [21] and found no common biomarkers for the two diseases. Therefore, the cause of the co-occurrence of the two diseases in this patient is unclear. This patient developed an intestinal fistula after laparoscopic surgery, and we analysed the possible factors as follows: (1) Intraoperative pulling could have led to damage to the intestinal canal. The patient was found to have multiple intestinal fistulas upon dissection, but some of the intestinal fistulas could have resulted from pulling during nonlaparoscopic repair of the intestinal canal; however, most fistulas were less likely to be caused by external physical stimulation. (2) The patient experienced a paroxysm; the release of specific factors, such as TNF-α, IL-1β, IL-6, and TGF-β, in the body after the stress of surgery could have accelerated the Crohn’s disease paroxysm and resulted in an intestinal fistula. During the formation of the vesicovaginal fistula in the patient, there was a transient increase in abdominal pain at the time of cystoscopic biopsy, and it is possible that the release of these pathogenic factors in the body had already been initiated or accelerated at this time. (3) After repair of the intestinal fistula and reappearance of the small intestinal fistula with a large amount of bloody stool and abdominal bleeding, the disease continued to progress, which is a highly unfavourable clinical finding and portends the ultimate outcome of the patient.

## Conclusion

This was a regrettable case. There are several points regarding the diagnosis of this patient worth summarizing. From the urologist’s point of view, the patient underwent a cystoscopic biopsy that revealed a vesicovaginal fistula. Second, the patient had experienced abdominal pain for more than 10 years, and although the patient reported relief with acid suppressants, she also had irregular stools, decreased haematocrit and weight loss, which were key factors that were not considered by the urologist. The patient was young but had a 10-year history of gastrointestinal disorders. No consultation with an enterologist was sought prior to surgery, which was a diagnostic deficiency. This case suggests that healthcare providers need to be aware of changes in the urinary system in patients with Crohn’s disease and consider gastrointestinal clinical manifestations in patients with cystitis glandularis.

## Data Availability

All data generated or analysed during this study are included in this published article.
